# Efficacy of complete laparoscopic ileal augmentation cystoplasty for the treatment of low bladder capacity and compliance: a case series

**DOI:** 10.1186/s12894-022-01008-5

**Published:** 2022-04-12

**Authors:** Chao Yang, Xin Chen, Yi Wang, Lu Fang, Wei Sun, Liangkuan Bi, Dexin Yu

**Affiliations:** Department of Urology, The Second Affiliated Hospital of Anhui Medical University, Hefei, 230032 Anhui China

**Keywords:** Safety and efficacy, Complete laparoscopic, Ileal augmentation cystoplasty, Treatment, Low bladder capacity and compliance

## Abstract

**Objectives:**

To investigate the safety and efficacy of complete laparoscopic ileal augmentation cystoplasty for the treatment of low bladder capacity and compliance.

**Methods:**

The clinical data of 13 patients with low bladder capacity and compliance were retrospectively analyzed. Therapeutic efficacy was evaluated at follow-up. The Clavien system was used to evaluate the severity of postoperative complications.

**Results:**

All 13 operations were successfully completed laparoscopically. The operation duration was 140–248 min (average: 189.9 ± 29.6 min), the time to postoperative recovery of bowel function was 1–10 days (average: 2.9 ± 2.3 days). There were 4 cases of grade I complications and 1 case of grade II complications (i.e., paralytic ileus caused by urinary leakage from the anastomosis of the augmented bladder). Cystography showed that the morphology of the bladder was close to normal, and the maximum safe capacity and compliance of the bladder were significantly increased [103.8 ± 16.6 mL and 332.3 ± 20.5 mL, p < 0.01; 7.0 ± 1.3 mL/cm H_2_O and 32.4 ± 2.1 mL/cm H_2_O, p < 0.01]. All patients were able to urinate spontaneously after catheter removal.

**Conclusions:**

Complete laparoscopic ileal augmentation cystoplasty is a safe and feasible treatment for low bladder capacity and compliance, and has the advantages of less trauma, less bleeding, faster recovery of intestinal function, and fewer postoperative complications. This treatment effectively increases bladder capacity, protects upper urinary tract function, and improves patient quality of life, and thus warrants clinical application.

**Supplementary Information:**

The online version contains supplementary material available at 10.1186/s12894-022-01008-5.

## Introduction

Under normal conditions, the elasticity and neuromodulation of the detrusor muscle maintain good compliance and stability of the bladder, thereby maintaining low pressure in the bladder for storage of urine. Low bladder capacity and compliance caused by neurogenic bladder, tuberculous bladder contracture, and interstitial cystitis is the primary mechanism leading to upper urinary tract damage and diminished quality of life. Bladder augmentation can reduce bladder pressure while also improving bladder capacity, making it the best procedure for treating low bladder capacity and compliance. Due to the difficulty of this operation, open surgery is currently in wide use for ileal augmentation cystoplasty in China. Our department uses complete laparoscopic ileal augmentation cystoplasty to treat low bladder capacity and compliance with good outcomes, as reported here.

## Methods

### Clinical data

The clinical data of 13 patients with low bladder capacity and compliance caused by neurogenic bladder, tuberculous bladder contracture, interstitial cystitis, and other benign diseases who underwent laparoscopic ileal augmentation cystoplasty at the Second Affiliated Hospital of Anhui Medical University between January 2018 and June 2021 were retrospectively analyzed. Of the 13 patients, 7 were male and 6 were female, and they ranged in age from 33 to 69 years (average: 51.8 years). There were 3 cases of neurogenic bladder (2 cases who suffered from diabetes mellitus had a low compliance bladder, while the rest case had an unexplained detrusor overactivity), 7 cases of (tuberculous) bladder contracture, and 3 cases of interstitial cystitis. The disease course ranged from 1 to 16 years (average: 5.2 years). All patients experienced concomitant urinary tract symptoms to varying degrees, such as frequent or urgent urination, with urination once every five minutes or urge incontinence in severe cases. All patients routinely received anti-cholinergic treatment prior to the operation and only received operation when they were refractory to the drug and refused botulinum toxin type A (BTX-A) injection therapy. Preoperative urodynamic testing indicated high bladder pressure during the storage phase (> 40 cm H_2_O). The bladder capacity was decreased, and the detrusor muscle was overactive, thus decreasing bladder compliance (≤ 10 mL/cm H_2_O) [1]. Evaluation of upper urinary tract function included urinary system ultrasound, computed tomography (CT), or magnetic resonance urography (MRU). There was ureteropelvic distension and fluid accumulation in 14 kidneys in 8 patients. Cystography revealed 4 cases of vesicoureteral reflux (1 case of bilateral reflux, 1 case of simple left-sided reflux, 2 cases of simple right-sided reflux). The grade of reflux was based on the international reflux scoring classification standards [2]: 1 case of grade II and below and 3 cases of grade III and above. Preoperative blood creatinine was 109–241 μmol/L (average: 161.7 μmol/L), and the glomerular filtration rate (GFR) was 30.32–72.21 mL^−1^ min 1.1.73 m^−2^ (average: 47.9 mL^−1^ min-1.1.73 m^−2^).

### Surgical procedure

**Fig. 1:**
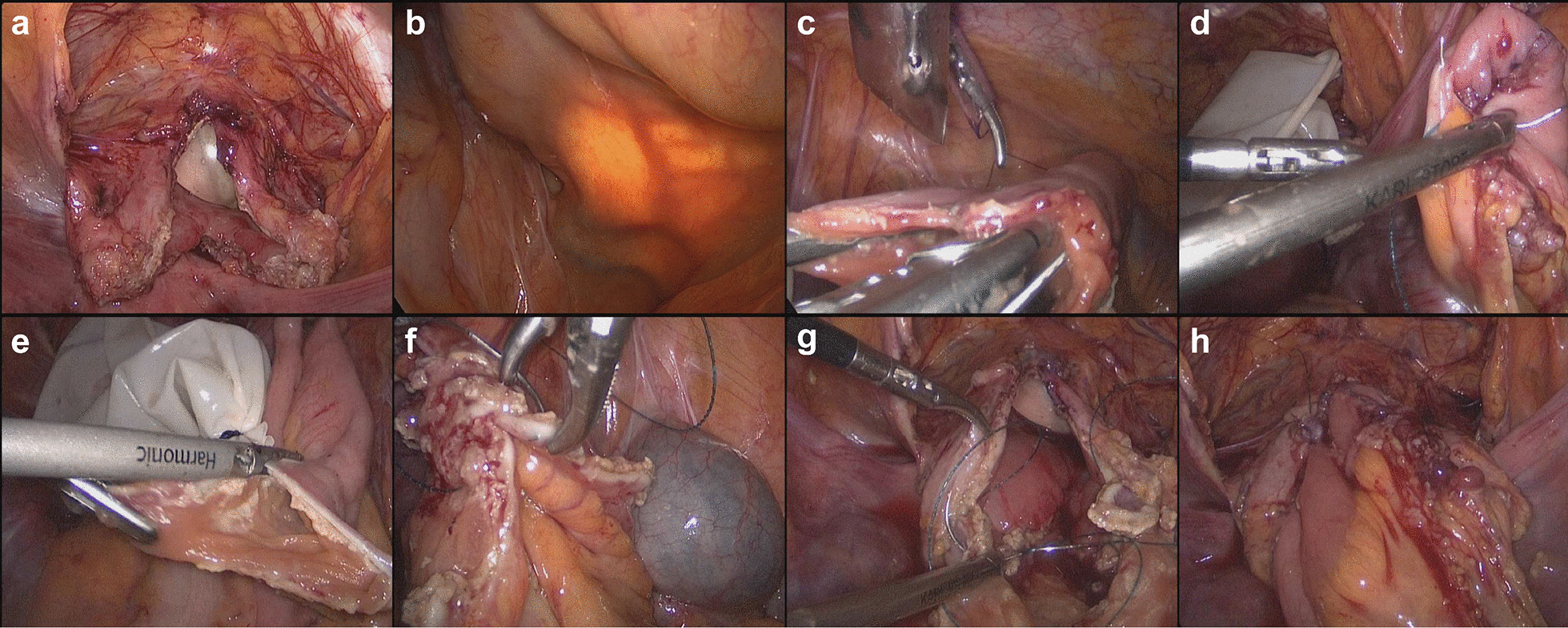
Intraoperative images of complete laparoscopic ileal augmentation cystoplasty. **A** resection of the apical anterior wall of the bladder; **B** confirmation of mesenteric blood vessels under laparoscopic light source; **C** side-to-side anastomosis of the intestine with the EC60 stapler; **D** continuous suture with barbed suture to restore bowel continuity; **E** splitting the bowel longitudinally on the medial side of the mesangium; **F** continuous suture of the inner wall of the intestinal segment with barbed suture; **G** suturing the ileal pouch to the bladder wall; **H** ileum and bladder after reconstruction

Routine bowel preparation was performed the day before surgery. During the operation, the head was placed in a 30° downward position. After satisfactory anesthesia was achieved, disinfection, draping, and indwelling urinary catheterization were performed. Pneumoperitoneum was created by inserting a pneumoperitoneum at a site two finger-widths above the umbilicus. A 10-mm cannula was inserted as an observation port. Under direct vision, 12-mm cannulas were inserted through the right and left rectus abdominis, 2 cm below the umbilicus. A 5-mm cannula was inserted above the right anterior superior iliac crest. Then, 50–100 mL of water was injected into the bladder, and the apex of the bladder wall was excised horizontally to partially remove the contracture of the bladder tissue so that the apex of the bladder could be fully expanded to form a bowl-like morphology for anastomosis (Fig. 1A). Under a laparoscopic light source, 30 cm of the terminal ileal loop was excised about 15 cm away from the ileocecal region, and a linear closure (EC60 stapler) was used to extend the upper and lower ends into the distal and proximal ileal lumen with side-to-side anastomosis of the bowel (Fig. 1B, C). Next, the ileal incision was continuously sutured with a 3-0 barbed suture to restore bowel continuity (Fig. 1D). The resulting intestinal loop was folded into a U shape, the intestine was split longitudinally at the mesangial margin and close to the mesangium (Fig. 1E). The intestinal mucosa was scrubbed with iodophor gauze, and the inner wall of the intestinal segment was continuously sutured with a barbed suture (Fig. 1F). The lowest point of the U-shaped intestinal segment was fixed to the highest point of the apical wall of the bladder was absorbable suture, and then the double-needle barbed suture was used to suture the intestinal segment and the bladder clockwise to 6 o'clock and counterclockwise to 12 o'clock (Fig. 1G, H). The 3 patients with vesicoureteral reflux of grade III and above underwent laparoscopic ureterovesical reimplantation (nipple method) at the same time, of which the ureters were implanted in the neobladder wall in two patients with tuberculosis and in the original bladder wall in one patient with interstitial cystitis. The distal of the ureter was pulled out of body through the trocar hole and formed into the nipple after tailoring and suturing. After that, a double J stent was inserted into the ureter. Then the ureter was put back into the peritoneal cavity and the ureteral distal was anastomosed to the bladder mucosa. Injection of water into the bladder did not reveal any obvious leakage of urine. A drainage tube was placed in the pelvic cavity and the operation was completed.

### Grading of postoperative complications and follow-up

The Clavien system was used to grade the severity of complications within 30 days after the operation to evaluate safety [3]. Outpatient follow-up examinations were conducted at 3, 6, 12, 18, and 24 months after the operation. Follow-up examinations included: complete blood count, urinalysis, renal function tests, blood gas analysis, voiding diary, B-scan ultrasound or CT of the urinary system, residual urine testing, and examinations such as cystoscopy and urodynamic testing.

### Statistics

SPSS 20.0 software was used for data entry and statistical analysis. Results are expressed as x ± s. One-way and multi-way analyses of variance were used to analyze whether there were differences in values at different time points. Differences with p < 0.05 were considered statistically significant.

## Results

The operation was completed successfully in all 13 patients without conversion to open surgery. The operation duration was 140–248 min (average: 189.9 ± 29.6 min); the intraoperative blood loss was 20–200 mL (average: 58.5 ± 50.0 mL); the time to postoperative recovery of bowel function was 1–10 days (average: 2.9 ± 2.3 days); the duration of postoperative hospital stay was 6–25 days (average: 8.8 ± 5.0 days); the time to catheter removal was 11–20 days (average: 13.5 ± 2.4 days). One patient developed a postoperative ileovesical anastomotic leak complicated with paralytic ileus: the patient recovered after conservative treatment, including gastrointestinal decompression, adjustment and cleaning of the catheter and pelvic drainage tubes, and intravenous nutritional support. There were 4 cases of grade I complications; all were cured after symptomatic treatment. There were no cases of ileal anastomotic leaks, mechanical bowel obstruction, severe abdominal infection, or other complications (Table 1).

**Table 1:** Baseline and surgical parameters of patients

No	Sex	Age (years)	BMI	Diagnosis	Ureterovesical reflux	Operation duration (min)	Intraoperative blood loss (mL)	Time to recovery of bowel function (d)	Duration of hospital stay (d)	Time to catheter removal (d)	Postoperative complications
1	M	33	27.7	Urinary tract tuberculosis	None	140	20	3	8	13	None
2	F	47	21.23	Interstitial cystitis	None	161	30	2	7	12	Infection at incision site
3	M	55	28.52	Urinary tract tuberculosis	Right	193	100	4	6	11	None
4	M	69	22.43	Urinary tract tuberculosis	Bilateral	248	200	2	6	16	None
5	F	60	28.44	Interstitial cystitis	None	246	30	2	6	15	None
6	M	65	23.44	Neurogenic bladder	Right	185	30	10	25	20	Ileovesical anastomotic leak
7	F	35	19.98	Neurogenic bladder	None	190	30	1	8	13	None
8	F	47	23.56	Urinary tract tuberculosis	None	204	50	2	9	12	Partial adhesive small-bowel obstruction
9	F	52	22.37	Neurogenic bladder	None	186	40	2	7	13	None
10	M	58	24.18	Urinary tract tuberculosis	None	159	30	3	9	12	Fever
11	F	49	25.03	Interstitial cystitis	Right	210	80	4	7	14	None
12	M	62	23.65	Urinary tract tuberculosis	None	204	30	2	8	13	Partial adhesive small-bowel obstruction
13	M	41	27.32	Urinary tract tuberculosis	None	163	90	1	6	12	None

**Fig. 2:**
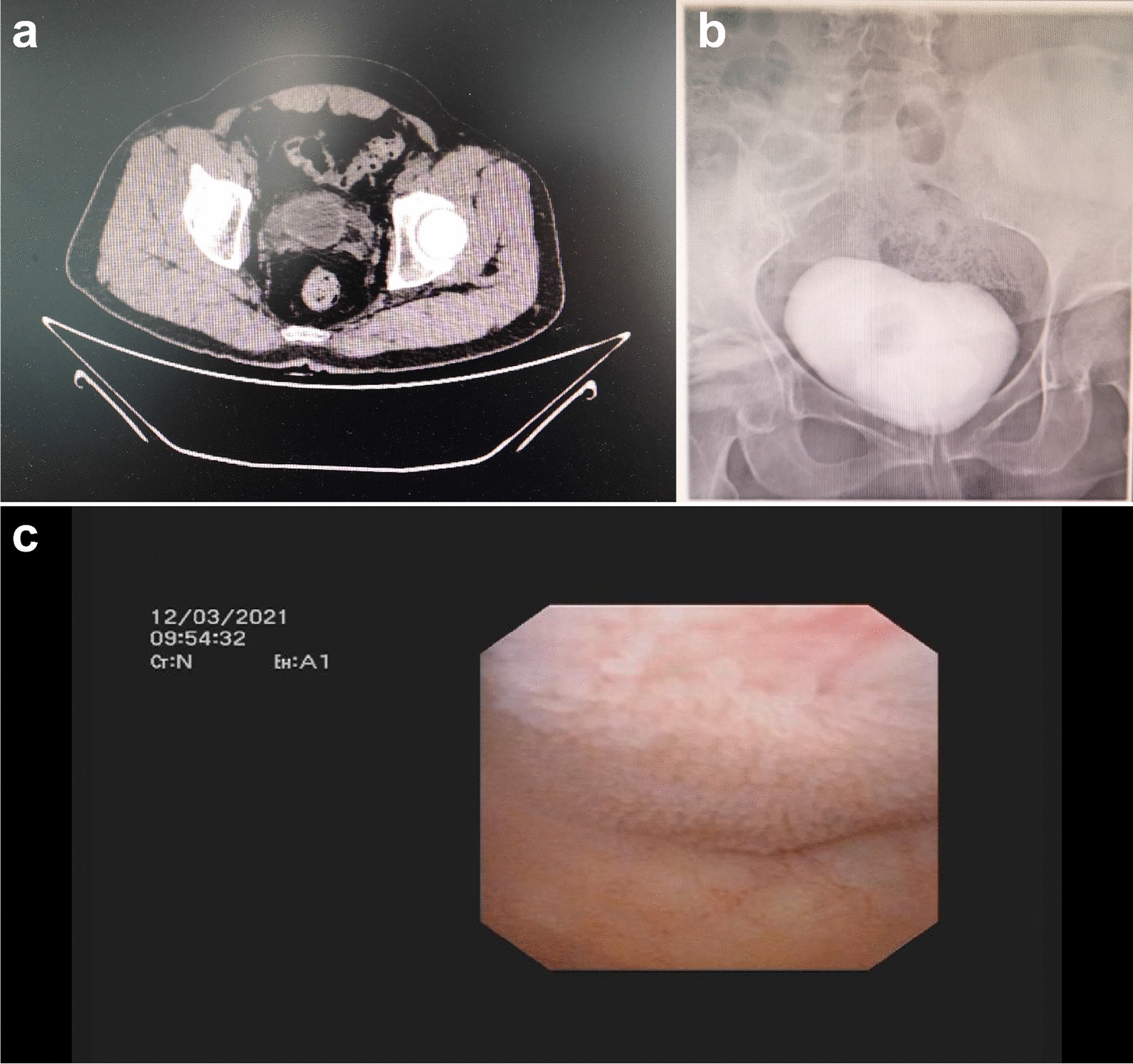
Bladder contracture imaging examination and postoperative cystoscopy results. **A** pre-op pelvic CT indicated bladder contracture with bilateral ureteral dilation; **B** cystography at 6 months post-op indicated a significant increase in bladder capacity; **C** cystoscopy at 6 months post-op revealed a red color in the mucosa of the ileum and bladde wall, with no urolithiasis at the anastomosis

The postoperative follow-up period was 5–24 months (average: 16.5 months). Serum creatinine and total glomerular filtration rate were improved (p < 0.05) (Table 2). Postoperative B-scan ultrasound and CT examination showed improvement of ureteropelvic distension and fluid accumulation in 8 patients. Cystography showed that the morphology of the bladder was close to normal and that there was no vesicoureteral reflux (Fig. 2A, B). Postoperative cystoscopy indicated good growth of the ileovesical wall, and there was no urolithiasis at the anastomosis site (Fig. 2C). The maximum safe capacity and compliance of the bladder were significantly increased [103.8 ± 16.6 mL and 332.3 ± 20.5 mL, p < 0.01; 7.0 ± 1.3 mL/cm H_2_O and 32.4 ± 2.1 mL/cm H_2_O, p < 0.01] (Table 2). All patients were able to urinate spontaneously after catheter removal. Three patients with neurogenic bladder still had urinary incontinence, but frequency was reduced [6.3 ± 0.6 times/day and 2.7 ± 0.6 times/day, p = 0.01], and the urine flow rate and residual urine of the remaining patients were significantly improved [7.4 ± 1.3 mL/s and 20.0 ± 8.9 mL/s, p = 0.01; 36.0 ± 10.8 mL and 17.0 ± 11.8 mL, p = 0.025]. Single urine volume and maximum urine flow rate were significantly increased, and residual urine volume decreased (Table 3).

**Table 2:** Comparison of various indicators in the 13 ileal augmentation cystoplasty cases before and after operation

Time	Creatinine (µmol/L)	Total GFR (ml^−1^ min^−1^ 1.73 m^−2)^	Maximum safe bladder capacity (mL)	Bladder compliance (mL/cm H_2_O)
Pre-op	161.7 ± 44.7	47.9 ± 13.5	103.8 ± 16.6	7.0 ± 1.3
3 months post-op	122.2 ± 30.3^#^	62.6 ± 16.2^#^	302.5 ± 22.3^*^	29.1 ± 2.8^*^
6 months post-op	116.5 ± 25.0^#^	64.5 ± 13.5^#^	318.5 ± 19.5^*^	31.0 ± 2.7^*^
1 year post-op	114.6 ± 22.8^#^	65.1 ± 12.6^#^	332.3 ± 20.5^*^	32.4 ± 2.1^*^

All data are expressed as mean ± SD; ^#^p < 0.05, ∗p < 0.01 compared to pre-op

**Table 3:** Comparison of indicators related to voluntary urination in 10 cases and urinary incontinence in 3 cases before and after operation

Time	Daily number of urinations (d)	Single urine volume (mL)	Maximum urine flow rate (mL/s)	Residual urine volume (mL)	Urinary incontinence	
					Times (d)	Pads used
Pre-op	22.5 ± 4.5	67.0 ± 9.5	7.4 ± 1.3	36.0 ± 10.8	6.3 ± 0.6	4.3 ± 0.6
3 months post-op	11.8 ± 5.5^a^	190.0 ± 84.5^a^	16.6 ± 7.4^a^	19.8 ± 10.6^a^	4.7 ± 0.6^a^	3.7 ± 0.6
6 months post-op	9.6 ± 4.4^a^	207.0 ± 91.9^a^	18.2 ± 8.1^a^	18.5 ± 9.6^a^	4.3 ± 0.6^a^	3.3 ± 0.6
1 year post-op	8.4 ± 3.8^a^	224.0 ± 99.0^b^	20.0 ± 8.9^a^	17.0 ± 11.8^a^	2.7 ± 0.6^a^	2.3 ± 0.6^a^

All data are expressed as mean ± SD; ^a^p < 0.05, ^b^p < 0.01 compared to pre-op

## Discussion

Low bladder compliance can result from various bladder-derived diseases, radiation cystitis, interstitial cystitis, (tuberculous) bladder contracture, bladder schistosomiasis, among others. The principle of treatment is primarily to safeguard renal function, and thus to improve patient quality of life. Measures that can effectively reduce intravesical pressure in the treatment of low bladder compliance include suprapubic bladder fistula, clean intermittent self-catheterization, urinary diversion, and various types of bladder augmentation. The choice of treatment option depends on the physician's experience and the patient's wishes. Intestinal augmentation cystoplasty is a good choice for the treatment of low bladder compliance and is also the current gold standard internationally [4]. The safety and efficacy of augmentation cystoplasty with vascularized gastrointestinal tract have been previously confirmed. The ileum, colon, ileocecum, and stomach can all be used as material for bladder augmentation [5, 6], but ileal and sigmoid augmentation cystoplasty are the most common.

Due to the difficulty of ileal augmentation cystoplasty and its complex steps, open surgery has predominantly been used in the past, both in China and abroad. Although open surgery has clear curative efficacy, it involves relatively high surgical trauma, has a long postoperative recovery period, and is prone to many complications [7]. In recent years, more minimally invasive and effective methods are being investigated in clinical practice with laparoscopic techniques becoming more widespread. There have been many foreign reports of laparoscopic (or robot-assisted) intestinal augmentation cystoplasty [8, 9]. Since January 2018, our department has treated 13 cases of low bladder capacity and compliance with ileal augmentation cystoplasty, and we summarized our experience as follows:

Complete laparoscopic ileal augmentation cystoplasty can minimize the impact of surgical stress on bowel function. Laparoscopy provides a clear magnified view that facilitates identifying the location of the mesenteric vessels. In addition, when cutting the terminal ileum, another laparoscopic lens is inserted through the operation port. The route of the mesenteric vessels can be clearly observed under the light source, thereby reducing damage to the intestinal blood supply, which is conducive to the recovery of bowel function. A complete laparoscopic operation can prevent exposure of the bowel outside the body, reduce interference with the bowel, and reduce the occurrence of postoperative intestinal adhesions and intestinal adhesive obstruction.

Fully laparoscopic restoration of bowel continuity using a linear cutting and closing device with a barbed suture is safe and effective. Linear closure (60 mm) was used to anastomose the distal and proximal ends of the ileum, and barbed sutures were used to close the end of the bowel incision to restore bowel continuity. The average duration of this procedure is about 15 min, which is much shorter than traditional manual suturing of the bowel. A wide anastomosis is conducive to early recovery of bowel function. The average postoperative time to normal eating was 2.9 ± 2.3 days. There were no cases of mechanical intestinal obstruction or intestinal fistula.

Fully laparoscopic manual suture of the ileal patch with barbed suture and cystoplasty is safe and economical and does not significantly increase operation duration. After cutting the intestinal segment, an ultrasonic knife is used to split the intestine longitudinally at the mesangial margin near the mesangial side. The inner wall of the intestinal segment is continuously sutured with a barbed suture to form a bladder patch. During the operation, 2 barbed sutures are used, and the average time for bladder pouch establishment is about 35 min, which is slightly longer than the method described by Qi et al. [10], involving an intestinal closure device to establish a bladder pouch (16 ± 3 min). However, the latter approach requires an electric knife to cut the bottom of the U-shaped closed intestinal segment, which is then anastomosed with the bladder wall.

Selecting the appropriate position and length of the ileum can significantly reduce the development of long-term complications. The ideal site for ileum harvesting is 25–40 cm away from the ileocecal valve, as secretion and absorption are weakest at this intestinal segment and the risk of postoperative metabolic disorders is minimal. The optimal ileum length has been reported in the literature to be about 20 cm. Although the ileum has a thin wall and good compliance, it greatly increases bladder capacity after detubularization. However, our study found that ileal augmentation cystoplasty using this length still results in a small effective bladder capacity, and compliance may be insufficient. Thus, we routinely cut about 30 cm of the ileum to establish the bladder pouch. Re-examination at 3 months post-op showed significant improvement of bladder compliance, and bladder volume did not increase progressively over time.

During ileovesical anastomosis, care should be taken to avoid stenosis of the ileovesical anastomosis. In the initial stages of bladder scar formation, the apex of the bladder contracts significantly, while the trigone and the neck are relatively unaffected [11]. During the operation, the apical anterior wall of the bladder should be opened to ensure sufficient width of the anastomosis. In patients with tuberculous bladder contracture, part of the apical wall of the bladder must be resected. At our hospital, doubly barbed sutures are used in all patients to suture the intestine and bladder wall left and right from the apex of the bladder, and the bladder pouch is sutured symmetrically to the apex of the bladder as much as possible, in order to ensure that the shape of the bladder is as normal as possible, thereby reducing the pressure in the bladder and improving its compliance.

Vesicoureteral reflux is one of the most common complications of low bladder compliance, with an incidence of 10–60%. Treatment of ureteral reflux depends on bladder capacity, pressure, and compliance. After ileal augmentation cystoplasty, the capacity of the bladder is increased and pressure is decreased, and in some cases ureteral reflux can disappear or improve spontaneously. In this cohort of cases, ureterovesical reimplantation was routinely performed for ureteral reflux of grade III and above, and one patient with grade II ureteral reflux only underwent ileal augmentation cystoplasty. Re-examination at 3 months post-operatively indicated less ureteropelvic distension and fluid accumulation in all cases compared to pre-operatively. The tunnel method has poor resistance to reflux since the ileum wall is thin, whereas the nipple method facilitates anastomosis and has good resistance to reflux, making it particularly suitable for ileal augmentation cystoplasty.

Close surveillance is necessary after ileal augmentation cystoplasty to prevent various complications. Cystoliths can occasionally develop, which may be associated with intestinal mucus accumulation or exposure of bladder sutures or staples from the linear closure in the bladder cavity [12]. Therefore, topical saline was used to flush the bladder daily during the perioperative period to prevent accumulation of intestinal mucus, and barbed thread was used to reconstruct the ileovesical pouch during the operation. The time to absorption was about 3–6 months, and urinary re-examination was performed at 3- and 6-months postoperatively. No cystoliths were found on color Doppler ultrasound or cystoscopy, and the probability of urolithiasis recurring later was significantly decreased. The intestinal mucosa of the augmented bladder reabsorbs chloride and acid (as ammonia), which can lead to water-electrolyte and acid–base imbalances. Postoperative surveillance must include dynamic blood gas analysis, and oral sodium bicarbonate should be administered as needed for preventive treatment.

Complete laparoscopic ileal augmentation cystoplasty is difficult due to the complex operation procedure and technical difficulties. Therefore, the surgeon must be proficient in laparoscopic separation and suture techniques. This preliminary investigation demonstrated that complete laparoscopic ileal augmentation cystoplasty is safe and feasible and has the advantages of less trauma, less bleeding, faster recovery of intestinal function, and fewer postoperative complications. This procedure effectively increases bladder capacity, protects upper urinary tract function, and improves patient quality of life, and thus warrants clinical application.

## Supplementary Information

**Additional file 1.** Patient characteristics and therapeutic information.

## Data Availability

The datasets used and/or analysed during the current study are available from the corresponding author on reasonable request.

## Abbreviations

CT: Computed tomography
MRU: Magnetic resonance urography
GFR: Glomerular filtration rate
